# Proline Scanning Mutagenesis Reveals a Role for the Flap Endonuclease-1 Helical Cap in Substrate Unpairing*

**DOI:** 10.1074/jbc.M113.509489

**Published:** 2013-10-14

**Authors:** Nikesh Patel, Jack C. Exell, Emma Jardine, Ben Ombler, L. David Finger, Barbara Ciani, Jane A. Grasby

**Affiliations:** From the Centre for Chemical Biology, Department of Chemistry, Krebs Institute, University of Sheffield, Sheffield S3 7HF, United Kingdom

**Keywords:** Circular Dichroism (CD), DNA Physical Chemistry, DNA Repair, DNA Replication, DNA-Protein Interaction, Enzyme Catalysis, Enzyme Mechanisms, Fluorescence, Fluorescence Correlation Spectroscopy, Nucleic Acid Enzymology

## Abstract

The prototypical 5′-nuclease, flap endonuclease-1 (FEN1), catalyzes the essential removal of single-stranded flaps during DNA replication and repair. FEN1 hydrolyzes a specific phosphodiester bond one nucleotide into double-stranded DNA. This specificity arises from double nucleotide unpairing that places the scissile phosphate diester on active site divalent metal ions. Also related to FEN1 specificity is the helical arch, through which 5′-flaps, but not continuous DNAs, can thread. The arch contains basic residues (Lys-93 and Arg-100 in human FEN1 (hFEN1)) that are conserved by all 5′-nucleases and a cap region only present in enzymes that process DNAs with 5′ termini. Proline mutations (L97P, L111P, L130P) were introduced into the hFEN1 helical arch. Each mutation was severely detrimental to reaction. However, all proteins were at least as stable as wild-type (WT) hFEN1 and bound substrate with comparable affinity. Moreover, all mutants produced complexes with 5′-biotinylated substrate that, when captured with streptavidin, were resistant to challenge with competitor DNA. Removal of both conserved basic residues (K93A/R100A) was no more detrimental to reaction than the single mutation R100A, but much less severe than L97P. The ability of protein-Ca^2+^ to rearrange 2-aminopurine-containing substrates was monitored by low energy CD. Although L97P and K93A/R100A retained the ability to unpair substrates, the cap mutants L111P and L130P did not. Taken together, these data challenge current assumptions related to 5′-nuclease family mechanism. Conserved basic amino acids are not required for double nucleotide unpairing and appear to act cooperatively, whereas the helical cap plays an unexpected role in hFEN1-substrate rearrangement.

## Teaser

**Background:** Human flap endonuclease-1, the prototypical 5′-nuclease, removes 5′-flaps by incising one nucleotide into duplex DNA using a double nucleotide unpairing mechanism.

**Results:** Alteration of the hFEN1 helical cap structure, but not removal of conserved basic residues, prevents substrate unpairing.

**Conclusion:** The hFEN1 helical cap is required for substrate rearrangement.

**Significance:** Mechanistic details of 5′-nuclease catalysis are crucial for understanding DNA replication and repair.

## Introduction

**FIGURE 1.**
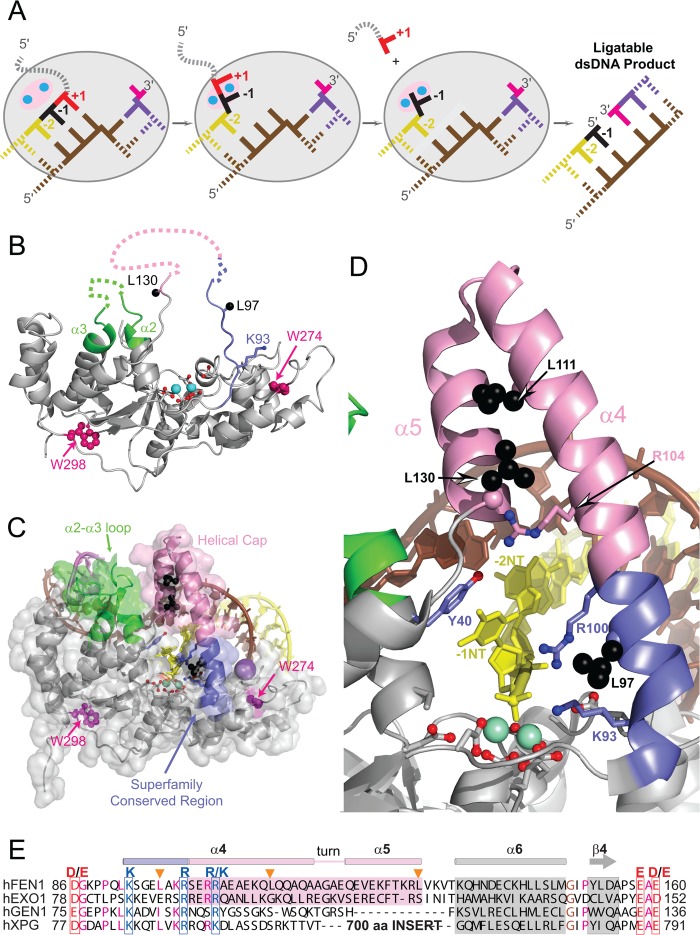
**hFEN1 structures, helical arch/cap structural transitions, and relationship to the other members of the 5**′**-nuclease superfamily.** *A*, schematic of an equilibrating double flap undergoing double nucleotide unpairing to allow hFEN1-catalyzed reaction, thereby generating a nicked DNA product. *B*, graphic representation of hFEN1 without substrate (1UL1:X) highlighting the disordered arch and α2-α3 loop. Superfamily conserved region of the arch (*blue*) and helical cap (*pink*) are shown with missing regions as *dotted lines*. Active site metal ions (*cyan*), carboxylates (*red*), and α2-α3 loop (*green*) are also highlighted. The side chains of the superfamily conserved basic residues Lys-93 and Arg-100 (*blue*), the two hFEN1 Trp residues (Trp-274, Trp-298; *dark pink*), and the positions of Leu residues that are mutated in this study (L97P, L111P, L130P, *black*) are shown (when defined in this structure). *C*, transparent surface representation of hFEN1 bound to product (3Q8K) with unpaired −1 nucleotide (*yellow*) with protein colored as in *B. D*, magnified view of the active site in 3Q8K and helical arch/cap with protein and residues colored as in *B* and *C*. hFEN1 conserved Tyr 40 (*blue*) is also shown. *E*, sequence alignment of hFEN1, human EXO1 (*hEXO1*), human XPG (*hXPG*), and human GEN1 (*hGEN1*) between Asp-86 and Asp-160 colored as in *B* highlighting conservation of basic amino acids and lack of conservation of the helical cap in hGEN1 and hXPG. The positions of the Leu residues mutated in this study are highlighted with an *orange triangle*.

Lagging strand DNA replication depends on the ability to process Okazaki fragments by removing single-stranded nucleic acid protrusions known as flaps that result from DNA polymerase-catalyzed strand displacement synthesis. In mammals, yeast, and some bacteria, FEN1 ^7^ elegantly catalyzes flap removal by recognizing a unique double-flap Okazaki fragment conformer with a 5′-flap of variable length, but only a single nucleotide 3′-flap (1–5). When subject to FEN1-catalyzed 5′-nucleolytic action, an incision is created one nucleotide into the double strand (see Fig. 1*A*). This results in a nicked DNA product that is immediately suitable for ligation, the last step of Okazaki fragment maturation. Similarly, DNA repair events that also depend upon DNA polymerase-catalyzed strand displacement synthesis, such as long patch base and ribonucleotide excision repair, have the same absolute requirement for the structure- and strand-specific nuclease FEN1.

In addition to flaps, other aberrant DNA structures also require 5′-nucleolytic action. Several of these aberrant structures are processed by alternative nucleases with sequence homology to FEN1; together, they form the FEN1 or 5′-nuclease superfamily (6). In humans, these include the DNA repair protein XPG that acts on bubble structures in nucleotide excision repair, EXO1 that acts on nicked or gapped DNAs in mismatch repair and recombination, and GEN1 that acts on Holliday junctions. An intriguing question raised by the superfamily is how such a diverse range of nucleic acid structures is processed with the exquisite substrate specificity necessary for each of the individual proteins using similar protein architecture.

Biochemical and structural studies of hFEN1 and other FEN1 family members have begun to reveal a mechanism for the action of 5′-nucleases (5–13). FEN1 family proteins all catalyze divalent metal ion-dependent phosphate diester hydrolysis one nucleotide into a duplex portion of their respective substrates. This reaction selectivity is achieved by double nucleotide unpairing of the duplex portion of substrate that abuts the DNA junction (see Fig. 1*A*). Unpairing allows the scissile phosphate diester bond to contact catalytic active site metal ions bound by a superfamily conserved carboxylate-rich active site (see Fig. 1, *B–D*).

FEN1 proteins have a structural feature known as the “helical arch” (α4-α5) that straddles the active site (see Fig. 1, *B–E*). The arch is partly disordered in substrate-free hFEN1 structures, as is the 3′-flap and duplex binding α2-α3 loop that packs against it when substrate is present (see Fig. 1*B*) (9, 14). The helical arch can be further subdivided into a region that contains two basic amino acids that are conserved in all 5′-nucleases (*i.e.* the base of α4, see Fig. 1, *D* and *E*) and a part that is not conserved in sequence alignments in all superfamily members (*i.e.* the C-terminal region of α4 and all of α5; ∼30 amino acids, see Fig. 1*E*). Here, we use the term helical arch to designate all of α4-α5, including the superfamily conserved region. We refer to the nonconserved 30 amino acids as the “helical cap” (6, 9, 15). When hFEN1 is bound to unpaired product, the nuclease domain of the protein is fully structured and the basic residues Lys-93 and Arg-100 contact the metal ion-bound 5′-phosphate monoester (see Fig. 1, *C* and *D*).

Possible roles for the helical arch and, in particular, its cap have received much attention (6, 8–10, 15–18). The helical cap is only present in 5′-nucleases hFEN1 and human EXO1 (hEXO1) that have a specificity for substrates that have 5′ termini-like flapped and gapped DNAs (6, 9, 15). The corresponding region in other family members is either much longer (XPG) or much shorter (GEN1) (see Fig. 1*E*). Biochemical data are consistent with a model where 5′-flaps are passed under the cap (15, 16). This would exclude processing of continuous DNA substrates such as bubbles and four-way junctions that have no 5′ termini, explaining the various substrate preferences within the superfamily (11, 15–19); however, this model is not universally accepted (10). It is also suggested that the helical cap may function to position superfamily conserved basic residues within the active site (6, 9, 16).

Here, to gain further insights into the role of the superfamily conserved α4-basic residues and cap secondary structure in hFEN1 in catalysis, we study the impact of mutations on substrate accommodation, unpairing, and hydrolysis. Mutation of superfamily conserved basic residues and introduction of helix breaking proline within the arch are severely detrimental to FEN1 function (3, 9). We demonstrate that these mutations compromise neither protein stability nor the ability to bind substrate. Moreover, all the proteins can still accommodate 5′-flaps in a manner consistent with threading this portion of the substrate underneath the cap. Instead, we find that the structural integrity of the cap, but not the superfamily conserved portion of the helical arch, is necessary to unpair hFEN1 substrates.

## EXPERIMENTAL PROCEDURES

### 

#### 

##### Enzymes

Constructs for the preparation of mutant proteins were prepared using the pET28b-hFEN1 plasmid (1) and the protocol outlined in the QuikChange® site-directed mutagenesis kit (Stratagene/Agilent). hFEN1 proteins were expressed and purified as described previously (9) except for L130P and K93A/R100A, which were only expressed for 4 h at 18 °C.

##### DNA Constructs

**FIGURE 2.**
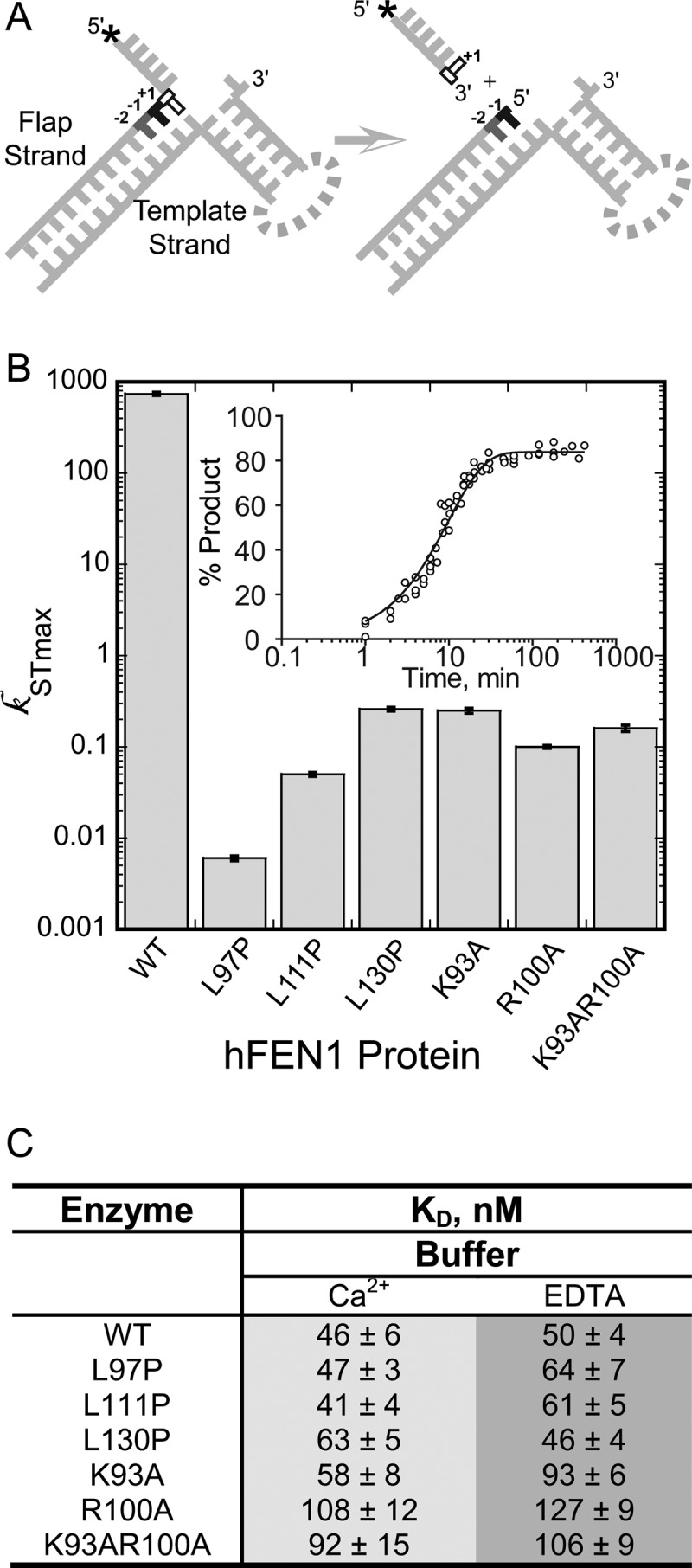
**Catalysis and substrate binding of arch hFEN-1 mutants.** *A*, schematic of the DF substrates used in this study illustrating the hFEN1-catalyzed reaction that occurs between the +1 and −1 nucleotides. Each static DF is composed of a flap and template strand, where 3′- and 5′-flaps are not complementary to template strand. Full sequence information is available in Table 1. In DF1, * = 5′-fluorescein; in DF2, * = 5′-biotin; in DF3, * = OH (unmodified); and in DF4, * = OH with both the −1 and −2 nucleobases as 2AP. *B*, maximal single turnover rates (*k_STmax_*) of WT and mutant hFEN1s (note log scale) with standard errors (S.E.) shown, measured at 37 °C and pH 7.5 using DF1. The *inset* shows example data for hFEN1 R100A fitted to Equation 1, and further details are under “Experimental Procedures.” *C*, substrate dissociation constants (± S.E.) in the presence of Ca^2+^ or EDTA for WT and mutant hFEN1s as determined by fluorescence anisotropy using DF1 at 37 °C and pH 7.5.

Oligodeoxynucleotides including those synthesized with 5′-fluorescein-CE-phosphoramidite (for DF1), 5′-biotin connected through a triethylene glycol linker (biotin-TEG) phosphoramidite (for DF2), or containing site-specific 2-amino purine (2AP) substitutions (for DF4) were purchased with HPLC purification from DNA Technology A/S. DNA concentrations were determined by UV absorbance at 260 nm using extinction coefficients generated using OligoAnalyzer 3.1. Heteroduplex double-flap (DF) substrates were prepared by heating the appropriate flap strand with the complementary template in a 1:1.1 ratio at 80 °C for 5 min in 50 mm Tris-HCl, pH 7.5, and 100 mm KCl with subsequent cooling to room temperature (see Fig. 2*A*). Sequences are given in Table 1.

**TABLE 1:**
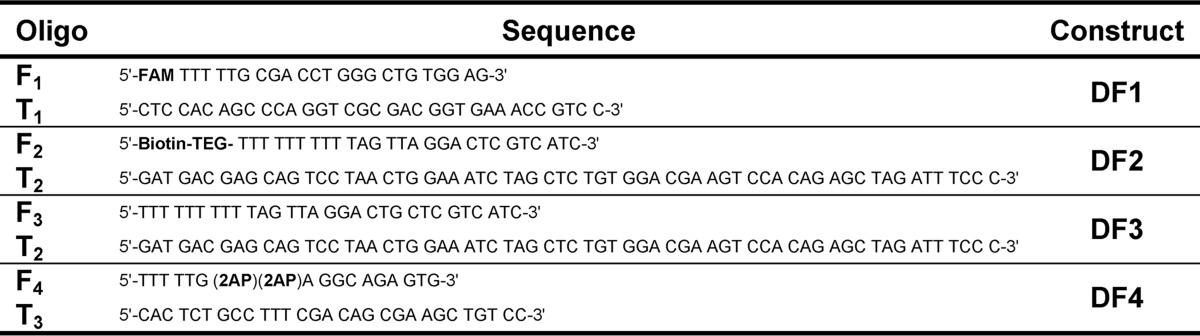
**Sequences of individual oligonucleotides used** **FAM,** 5′-fluorescein; **Biotin-TEG,** 5′-biotin connected through a tri(ethylene) glycol linker.

##### Maximal Single Turnover Rates

Maximal turnover rates of reaction were determined in triplicate using 2.5 nm DF1 and 1 μm hFEN1 or mutant protein in 55 mm HEPES, pH 7.5, 110 mm KCl, 8 mm MgCl_2_, 1 mm DTT (dithiothreitol), and 0.1 mg/ml BSA using rapid quench apparatus or manual sampling where appropriate at 37 °C as described (1). Reactions were analyzed by denaturing HPLC equipped with a fluorescence detector (Wave® system, Transgenomic) as described (1, 4, 20, 21). The data for the formation of product over time (*P_t_*) were modeled by nonlinear least squares regression in KaleidaGraph 4.0 using Equation 1 to determine the maximal first order rate constant (*k*) where *P*_∞_ is the amount of product at end point.

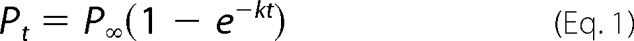


##### Fluorescence Anisotropy

Protein-DNA equilibrium dissociation constants were measured by fluorescence anisotropy using a Horiba Jobin Yvon FluoroMax-3® fluorometer with automatic polarizers. The excitation wavelength was 490 nm (excitation slit width 8 nm) with emission detected at 510 nm (10-nm-wide slit). Samples contained 2 mm EDTA or 10 mm CaCl_2_, 10 nm DF1, 55 mm HEPES, pH 7.5, 110 mm KCl, 0.1 mg/ml BSA, and 1 mm DTT at 37 °C. The first measurement was taken prior to the addition of protein with subsequent readings taken on the cumulative addition of enzyme, with corrections made for dilution. Data were modeled by nonlinear least squares regression in KaleidaGraph 4.0 using Equation 2,

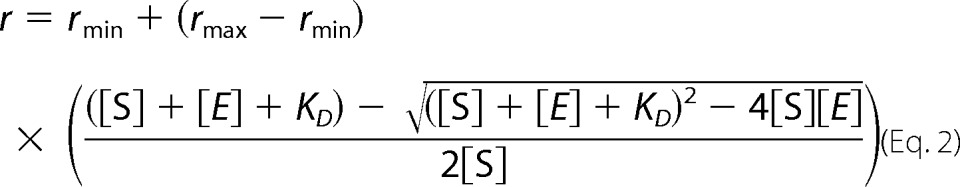
 where *r* is the measured anisotropy, [*E*] is the total protein concentration, [S] is the total DF1 concentration, *r*_min_ is the anisotropy of free DNA, *r*_max_ is the anisotropy of the DNA-protein complex, and *K_D_* is the equilibrium dissociation constant. Each measurement was independently repeated in triplicate.

##### Protein Circular Dichroism Spectroscopy

CD measurements of hFEN1 and mutants thereof were performed using a 0.1-cm path length cuvette and samples containing 3 μm protein in 1 mm potassium phosphate, pH 7.5, 30.7 mm (NH_4_)_2_SO_4_, 8 mm MgSO_4_, 0.1 mm EDTA, and 0.25 mm tris(hydroxypropyl)-phosphine. This buffer composition was used due to its low absorption in the near UV region (19, 22) and similar overall ionic strength to that used for determination of maximal single turnover rates of reaction. The CD spectra were collected between 190 and 260 nm in 1-nm steps with a response time and bandwidth of 8 s and 2 nm, respectively, at 20 °C using a JASCO J-810 CD spectrophotometer. Using the JASCO Spectra Analysis software (version 1.53.07), spectra were converted to mean residue molar ellipticity and smoothed by the means-movement method using a convolution width of five.

##### Fluorescence-detected Equilibrium Unfolding by Urea-induced Denaturation

Urea stock solution concentrations were determined by the method of Warren and Gordon (23). Prior to unfolding studies, proteins were subjected to size exclusion chromatography using 50 mm Tris, pH 8.5, 0.02% sodium azide, 1 mm DTT, and 100 mm KCl, and subsequently, concentration before determination of protein concentration by UV spectroscopy (*A*_280_, ϵ = 22,920 m^−1^ cm^−1^). Urea-induced denaturation of hFEN1 proteins (1 μm) was conducted in 50 mm Tris, pH 8.5, 0.02% sodium azide, 1 mm DTT with either 100 mm KCl, 8 mm MgCl_2_ or 120 mm KCl, 1 mm EDTA at 26 °C between 0 and 8 m urea in 0.5 m increments. Samples were prepared by removal of protein from the native protein stock and subsequent addition of an equal volume of denatured protein in buffer containing 8 m urea. At each urea concentration, the samples were allowed to attain equilibrium for 2 min, which was independently confirmed to be sufficient to reach equilibrium. Fluorescence measurements were performed on a Horiba Jobin Yvon FluoroMax-3® fluorometer using an excitation pulse at 295 nm and by collecting emission spectra using 10-nm slit widths between 300 and 500 nm. The final emission spectra were an average of five emission spectra.

**FIGURE 3.**
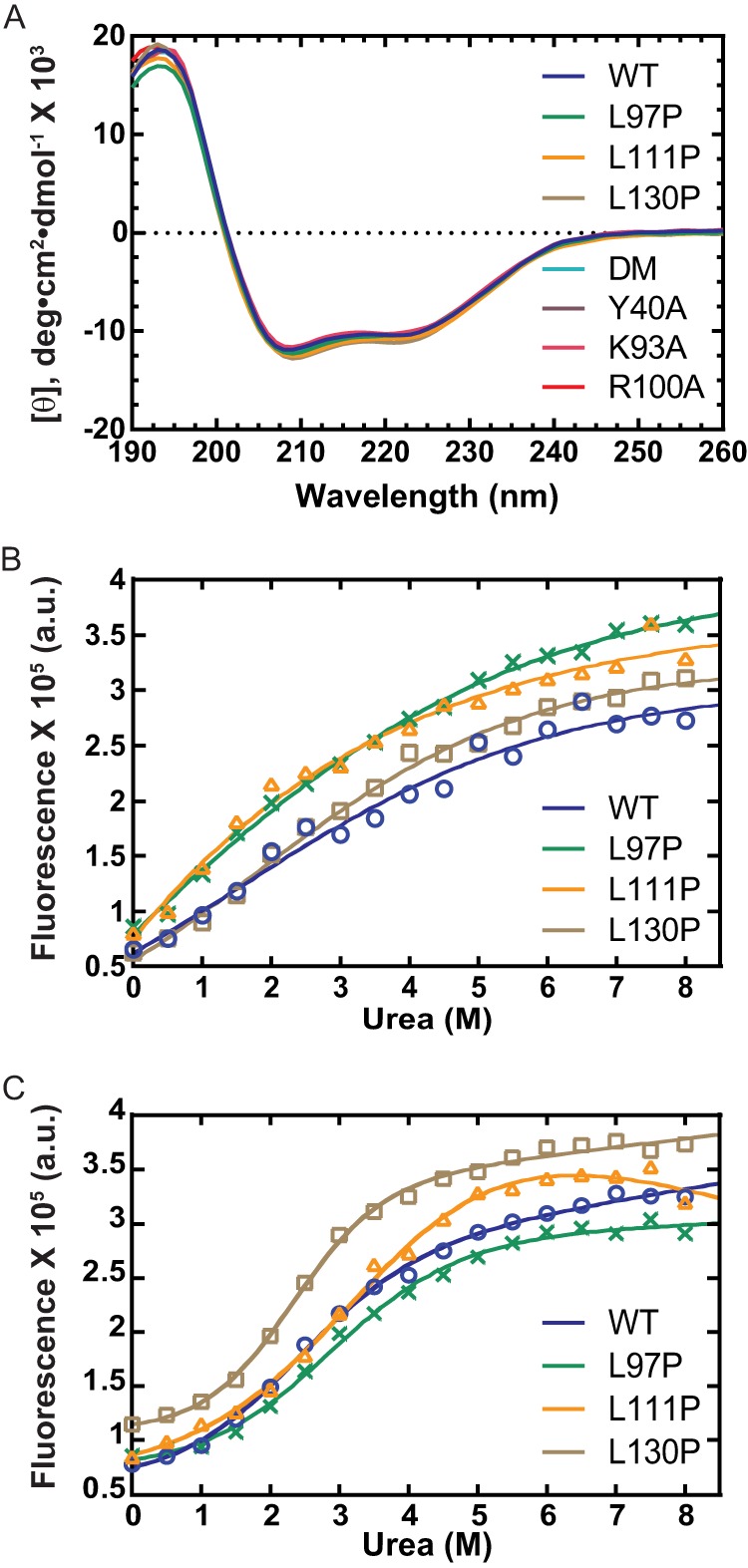
**Structure and stability of WT and mutated hFEN1s.** *A*, CD spectra of WT and mutated hFEN1 proteins (see *legend inset*) at pH 7.5 and 20 °C in Mg^2+^-containing buffer. *B*, urea denaturation curves of WT hFEN1 and proline mutants thereof (see *legend inset*) in the absence of metal ions and monitored by Trp fluorescence emission at 362 nm at 26 °C, pH 8.5. For clarity, data are presented with a cosmetic fit to Equation 3 without including pre- and post-transition base lines. (*a. u.* = arbitrary units) *C*, urea denaturation of WT hFEN1 and proline mutants thereof (see *legend inset*) in the presence of Mg^2+^ and monitored by Trp fluorescence emission at 362 nm at 26 °C, pH 8.5. Data are fitted to Equation 3 without including the pre-transition base-line slope to afford the midpoints of transition, [D]^50%^, which are reported in Table 2. Full details of conditions are provided under “Experimental Procedures.”

To obtain parameters for unfolding, the urea concentration dependence at the point of maximum change (362 nm) was fit to a two-state model appropriate for a monomer.

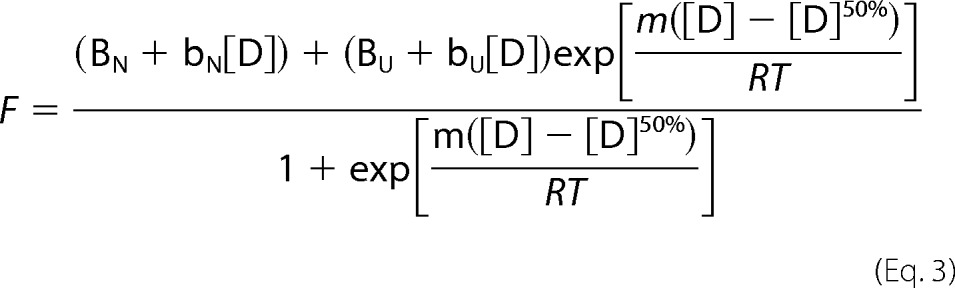
 Here, B_N_ and B_U_ are the base-line CD values for the native (N) and denatured (D) states, b_N_ and b_U_ are the slopes of the pre-and post-transitions, [D]^50%^ is the denaturant concentration at which the protein is 50% unfolded (24), and the *m*-value is the slope of the unfolding transition. Data were modeled by nonlinear least squares regression using KaleidaGraph 3.6 and Equation 3 with only five parameters as free variables in the fit. Moreover, due to the lack of pre-transition base line, this slope parameter was excluded from the fit. Parameters for the unfolding data in Fig. 3*B* were not derived given the lack of pre- and post-transition base lines; thus, the fit to the data is merely cosmetic. Δ*G*_D−N_^H_2_^O^^ values were calculated from *m*-values and [D]^50%^ using Equation 4.

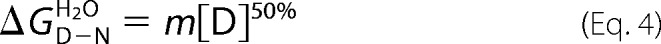


##### Streptavidin Blocking/Trapping Experiments

All FEN1-substrate binding reactions were prepared with 1 nm of the biotinylated substrate DF2 in 50 mm HEPES-KOH, pH 7.5, 100 mm KCl, 8 mm CaCl_2_, 0.25 mg/ml BSA, 2 mm DTT, and 5% glycerol. Where appropriate, the indicated protein (50 nm final concentration), streptavidin (Roche Diagnostics; 0.2 μg), and/or the nonbiotinylated competitor DNA (DF3; 250 nm final concentration) were added in specific orders to create blocked or trapped conditions. Blocked complexes refer to binding reactions that were prepared by incubating the substrate with streptavidin for 10 min at room temperature prior to the addition of the indicated hFEN1 protein. Trapped complexes refer to binding reactions that were prepared by incubating the substrate with the indicated hFEN1 protein for 10 min at room temperature before the addition of streptavidin to binding reactions. After the addition of the second component in both blocked and trapped binding reactions, the reactions were incubated for a further 10 min. For reactions where FEN1-substrate binding was challenged with DNA substrate lacking biotin, DF3 was added after the second incubation, and the mixture was allowed to incubate for 10 min at 37 °C.

##### Electrophoretic Mobility Shift Assay

The extent of binding in the various samples described above was assessed using a previously described electrophoretic mobility shift assay (EMSA) (16) in combination with the protocol of the chemiluminescent nucleic acid detection module kit (Thermo Scientific). Briefly, reaction mixtures (5 μl) were loaded onto native 6% polyacrylamide gels (29:1) containing 0.5× Tris-borate-EDTA while running at 200 V at room temperature and then further electrophoresed at 200 V until the bromphenol blue indicator in a separate well reached the bottom of the gel. The gels were then electroblotted onto Biodyne B nylon membranes (Thermo Scientific) at 4 °C in 0.5× Tris-borate-EDTA at 380 mA for 30 min using a Mini Trans-Blot® module (Bio-Rad). The membranes were removed from the blot sandwich and were exposed face-down to a transilluminator equipped with 312-nm bulbs for 15 min to cross-link all complexes. The blots were then incubated in nucleic acid blocking buffer (Thermo Scientific) for 30 min, and subsequently, incubated in 1:300 dilution of stabilized streptavidin-horseradish peroxidase (SA-HRP) in nucleic acid blocking buffer for 30 min. The blots were washed five times in 20 ml of 1× wash buffer and once in 20 ml of substrate equilibration buffer (Thermo Scientific). Enhanced chemiluminescence (ECL) detection was accomplished using chemiluminescence substrate (Thermo Scientific) and a ChemiDoc XRS system (Bio-Rad).

##### 2AP Exciton-coupled Circular Dichroism Spectroscopy (Low Energy CD)

Spectra were recorded on samples containing 10 μm DF4, 50 mm Tris-HCl, pH 7.5, 100 mm KCl, 1 mm DTT, either 10 mm CaCl_2_ or 10 mm CaCl_2_ + 25 mm EDTA and, where appropriate, 12.5 μm protein using a JASCO J-810 CD spectrophotometer (300–480 nm) at 20 °C as described (12). The CD spectra were plotted as Δϵ per mol of 2AP residue *versus* wavelength. Each measurement was independently repeated (typically in triplicate) and gave similar results.

## RESULTS

### 

#### 

##### Arch Mutations Inhibit hFEN1-catalyzed Reactions

To investigate the role of conserved and nonconserved regions of the helical arch (α4-α5) in the hFEN1 catalytic pathway, we used leucine to proline mutations to disrupt or alter the structure. Earlier, these same point mutations were isolated from an *in vivo* screen of dominant-negative hFEN1 mutants that were toxic to yeast (3). The proline mutations were either at the base of α4 (L97P) between the two superfamily conserved basic residues (Lys-93 and Arg-100) or in the helical cap region at the top of α4 (L111P) and end of α5 (L130P) (Fig. 1, *D* and *E*). A failure to properly position the α4 and/or α5 helices could alter the presentation of superfamily conserved basic residues Lys-93 and Arg-100 into the active site. We, therefore, studied mutations of these residues to alanine (K93A, R100A) and the double mutant (K93A/R100A) alongside the proline-mutated hFEN1s.

The maximal rates of reaction of the mutated hFEN1s were characterized under single turnover conditions (*i.e.* enzyme excess), using an optimal, nonequilibrating (*i.e.* static) double-flap substrate, DF1 (Fig. 2, *A* and *B*, and Table 2). In line with their strict conservation throughout the 5′-nuclease superfamily, replacement of either Lys-93 or Arg-100 with alanine has previously been shown to be detrimental in several FEN1 family proteins (9, 10, 25). Here, under maximal single turnover conditions, removal of either of the basic side chains decreased the rate of reaction by factors of ∼2000-fold (K93A) and 7000-fold (R100A), respectively (Fig. 2*B*). Interestingly, the magnitudes of the decreases in rate of reaction relative to WT hFEN1 were much greater under maximal single turnover conditions than when the catalytic efficiencies (*k*_cat_/*K_m_*) of the enzymes were measured (9). This likely arises because, at low concentrations of substrate (i.e. *k*_cat_/*K_m_* conditions), the rate of the wild WT hFEN1 reaction is rate-limited by encounter of enzyme and substrate and not by the proceeding steps (25). The double mutation K93A/R100A also decreased the rate of reaction by 3 orders of magnitude, producing a similar rate constant to R100A.

**TABLE 2:** **Denaturation parameters at 25 °C for hFEN1 and proline mutants** Parameters were calculated fitting raw fluorescence data to a 2-state unfolding model in urea with sloping baselines (Equations 3 and 4).

Sample	[D]^50%^ ± S.E.*^a^*	*m*-value ± S.E.*^a^*	Δ*G*^H_2_O^ ± S.E.*^a^*
	*m*	*kcal mol*^−*1*^ *m*^−*1*^	*kcal mol*^−*1*^
Wild type	2.26 ± 0.12	0.66 ± 0.08	1.49 ± 0.20
L97P	2.80 ± 0.17	0.63 ± 0.09	1.76 ± 0.27
L111P	3.40 ± 0.27	0.50 ± 0.07	1.70 ± 0.27
L130P	2.27 ± 0.07	0.92 ± 0.09	2.10 ± 0.21

*^a^* S.E. is the standard error of the fit.

Unsurprisingly, the mutation L97P, which is located between the two 5′-nuclease superfamily conserved basic residues, was highly detrimental to catalysis, drastically reducing the rate of reaction >100,000-fold. L97P is by far the most catastrophic point mutation of hFEN1 studied to date, and its impact cannot be accounted for solely by preventing participation of the key basic residues in catalysis. As L111P and L130P mutations are contained in the helical cap region of the protein that are not superfamily conserved, they were expected to have less of an impact on the rate of reaction. Nevertheless, L111P was also severely detrimental to the reaction, decreasing the maximal rate by a factor of ∼15,000-fold. Similarly, L130P decreased the rate constant ∼3,000-fold, further underscoring the importance of cap structural integrity to hFEN1-catalyzed reactions. Furthermore, the severities of rate reduction correlate with the relative severities of the reported dominant-negative phenotypes of these mutations upon overexpression in yeast (3).

##### Arch Mutations Do Not Alter Substrate Affinity

To examine the impact of the arch mutations on substrate binding, the dissociation constants of the respective proteins were measured by fluorescence anisotropy using DF1, which was synthesized with a 5′-fluorescein label (Fig. 2, *A* and *C*, and Table 1). In the presence of EDTA, the WT protein and all mutants had similar dissociation constants, showing only 2-fold variation. Calcium ions do not support hFEN1 catalysis and are a competitive inhibitor of FEN1 family reactions with respect to viable cofactor ions such as Mg^2+^, implying that they occupy the same active site positions (7, 26). However, even with occupation of the carboxylate-rich active site by Ca^2+^ ions, the dissociation constants of the WT and mutated hFEN1 complexes were relatively unchanged.

##### Proline Mutations Do Not Destabilize hFEN1

To investigate the impact of proline mutations on protein structure, we measured the CD spectra of the proteins in the presence of Mg^2+^ ions (Fig. 3*A*) and monitored unfolding of the proteins in the presence of urea (Fig. 3, *B* and *C*). The CD spectra of each of the mutated proteins closely resembled that of WT hFEN1, thereby demonstrating that no gross changes to the secondary structure of the proteins occurred. Equilibrium unfolding studies of the mutants were monitored by tryptophan fluorescence using urea as the denaturant. Data were fitted assuming two-state unfolding (Equation 3). The Trp residues of hFEN1 are buried in the main saddle region of the protein on either side of the β-sheet (Fig. 1, *A* and *B*). In the presence of EDTA, the denaturation curves of WT and mutant enzymes lacked pre- and post-transition base lines, which is indicative of the absence of cooperativity during the unfolding transitions (Fig. 3*B*). The addition of magnesium ions induced cooperativity in both the WT and the mutant equilibrium unfolding, which is consistent with the higher melting temperatures observed for WT samples monitored by differential scanning fluorometry under similar conditions (data not shown). In the presence of Mg^2+^ ions, the midpoints of transition for urea denaturation ([D]^50%^, Table 2) were similar or higher than the WT and mutated proteins, but were surprisingly low for an ∼40-kDa protein (27).

##### Arch Mutants Can Still Accommodate 5′-Flaps

Earlier work (15, 16) devised a methodology that allows the ability to properly accommodate the 5′-flap portion of substrates to be assessed. Here, “5′-flap accommodation” refers to positioning the 5′-flap in a manner that can readily proceed to reaction in the WT protein (15, 16). When streptavidin (SA) is added to 5′-biotinylated substrates in complex with WT hFEN1-Ca^2+^, the majority of the DNA can be trapped on the protein (15), indicating that the 5′-flap is trapped in a conformation that easily proceeds to reaction because the addition of Mg^2+^ ions results in reaction rates comparable with those of unmodified complexes. This is the case even when the trapped complex is challenged with excess unlabeled substrate before the addition of viable cofactor, demonstrating that trapped DNA cannot exchange. In contrast, when SA is conjugated to substrate before interaction with hFEN1-Ca^2+^, termed a blocked complex, reaction is drastically slowed. Blocked complexes readily dissociate in the presence of excess substrate, as do unmodified complexes (15, 16). These observations are interpreted as indicating that a nonblocked 5′-flap is able to pass under the helical cap and is able to be trapped in this state by SA, a conclusion supported by recent single-molecule studies (15, 16, 19).

**FIGURE 4.**
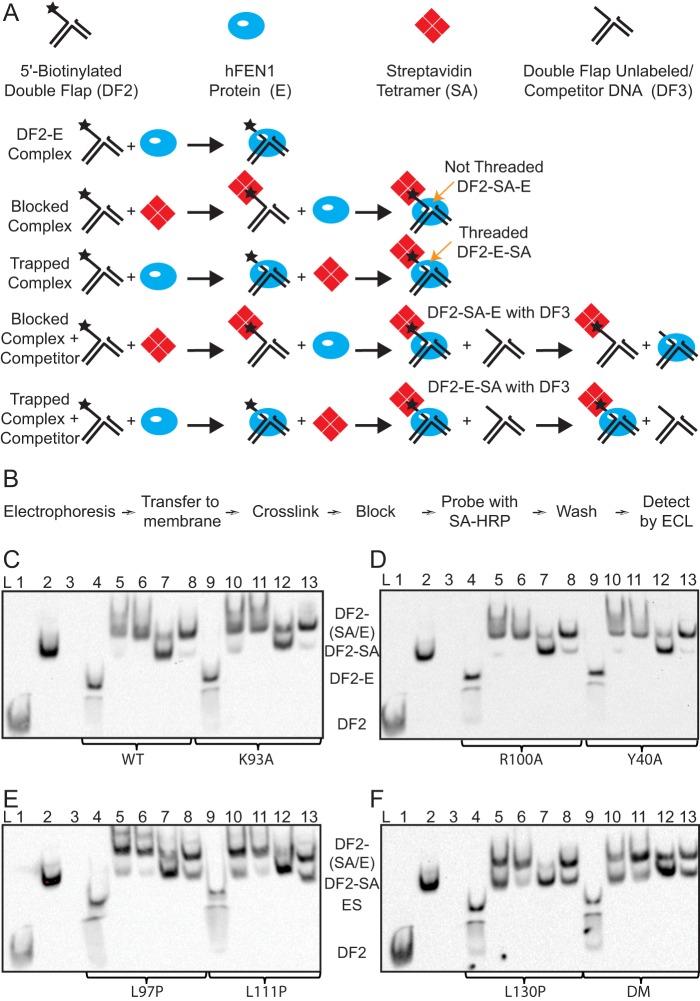
**5**′-**Flap accommodation assessment by EMSA.** *A*, schematic of procedure for the formation of complexes. Trapped complexes were assembled at 20 °C by the addition of SA to an E-DF2 complex, whereas blocked complexes involved the addition of E to SA-DF2. Competition experiments were carried out with 5-fold excess nonbiotinylated DF3 at 37 °C for 10 min. *B*, workflow diagram for detection of biotinylated oligonucleotides using SA-HRP conjugate. *C–F*, flap accommodation assessed by EMSA that was detected using the SA-HRP conjugate. *Lane 1*, DF2; *lane 2*, DF2-SA; *lane 3*, DF3 with SA (control, no signal observed without biotinylated DNA); *lanes 4* and *9*, DF2-E; *lanes 5* and *10*, blocked DF2-SA-E; *lanes 6* and *11*, trapped DF2-E-SA; *lanes 7* and *12*, blocked DF2-SA-E competed with DF3; *lanes 8* and *13*, trapped DF2-E-SA competed with DF3. *C*, WT hFEN1 (*lanes 4–8*) and K93A (*lanes 9–13*). *D*, R100A (*lanes 4–8*) and Y40A (*lanes 9–13*). *E*, WT L97P (*lanes 4–8*) and L111P (*lanes 9–13*). *F*, L130P (*lanes 4–8*) and K93A/R100A (*DM*) (*lanes 9–13*).

As the rates of reaction of the mutants studied here were very slow, we examined the ability of WT and mutated proteins to form blocked and trapped complexes with 5′-biotinylated substrate DF2 by EMSA (16). Thus, we prepared two types of enzyme-Ca^2+^-5′-SA-DF2 complexes (Fig. 4*A*). A blocked complex was formed by conjugation of SA to DF2 prior to the addition of the enzyme, preventing the passage of the 5′-flap underneath the cap. A second complex was formed by prior assembly of 5′-biotinylated hFEN1-Ca^2+^-DF2 complex followed by the addition of SA to trap the DNA in this conformer. In the trapped complexes, the 5′-flap could still have passed under the cap. Complexes were detected using an SA-HRP conjugate and enhanced chemiluminescence reagents (Thermo Scientific), which together detect biotinylated DNAs (Fig. 4*B*). Before developing this method, we rationalized that because some of our samples already contained biotinylated oligonucleotide conjugated to SA, the SA-HRP conjugate would be unable to detect any complexes of DF2 to which SA had been added prior to electrophoresis. Thus, we initially detected biotinylated DF2 complexes indirectly using an anti-SA antibody that was conjugated to HRP (Abcam). Although this method gave a high background, we were able to detect the various SA complexes in addition to the excess SA in samples to which it was added (data not shown). However, when we probed additional blots of the same samples with the SA-HRP conjugate, we were surprisingly able to observe all types of DF2 complexes in the context of a low background (Fig. 4, *C* and *F*). Despite the saturation of biotinylated oligonucleotides with SA in specific samples, the SA-HRP conjugate still detected all the biotinylated species after cross-linking/blotting. It seems likely that the cross-linking procedure results in denaturation of the SA bound to the biotinylated oligonucleotides in our electrophoresed samples.

Blocked (Fig. 4, *C–F*, *lanes 5* and *10*) and trapped (Fig. 4, *C–F*, *lanes 6* and *11*) SA complexes had similar electrophoretic mobility, but blocked and unmodified complexes were less stable under gel conditions. Blocked and trapped complexes were then challenged with excess unlabeled (*i.e.* no 5′-biotin moiety) competitor substrate, DF3 (Fig. 4, *C–F*, *lanes 7* and *12* (blocked) and *lanes 8* and *13* (trapped)) prior to electrophoresis. Regardless of hFEN1 protein, we found that blocked complexes were readily competed away, whereas the majority of trapped complexes were not displaced, as was observed earlier for the WT enzyme (15, 16). The degree of competition of trapped complexes does differ for some of the mutants, namely L97P, L111P, L130P, and K93A/R100A. We suspect that this indicates a change in the equilibrium constant between threaded and unthreaded forms of the enzyme-substrate complex. Nevertheless, all the mutated proteins could produce a trapped complex upon the addition of SA, suggesting that the ability to properly accommodate the 5′-flap substrate under the helical arch is not prevented.

##### Helical Cap Proline Mutants Cannot Unpair Substrates

**FIGURE 5.**
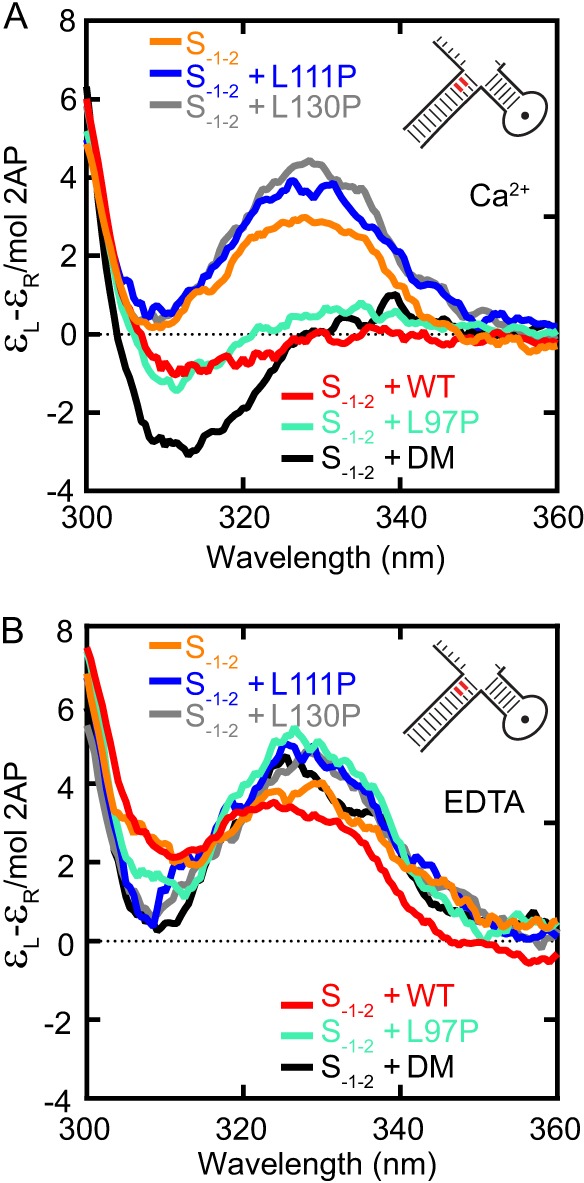
**Substrate unpairing by WT and mutated hFEN1 proteins.** *A* and *B*, low energy CD spectra of 2AP dimer containing DF4 and protein-DF4 complexes with divalent metal ions (10 mm CaCl_2_) (*A*) or without available divalent ions (10 mm CaCl_2_ + 25 mm EDTA) (*B*). Samples were buffered at pH 7.5, and measurements performed at 20 °C. DF4 is illustrated schematically with the position of 2APs (full sequence is shown in Table 1).

Because the proline mutations neither altered the ability to bind substrates nor impaired protein stability and each protein was able to accommodate a 5′-flap, we asked whether the mutant FENs could induce substrate conformational changes essential to the hFEN1 catalytic cycle (Fig. 1*A*). Previously, we monitored the ability of WT hFEN1-Ca^2+^ to unpair DNAs by reduction in the low energy CD signal resulting from the presence of a 2AP exciton pair in the substrate (12). Both protein and the natural nucleotides of the DNA have been shown to be transparent in this region of the CD spectrum. The adjacent 2APs were located at positions −1 and −2 in DF4. In the double nucleotide unpairing model, the −2 nucleotide will remain base-paired when complexed with hFEN1, whereas the −1 nucleotide will become extrahelical (Figs. 1*A* and 2*A* and Table 1). The addition of hFEN1 in the presence of Ca^2+^ ions, but not the presence of hFEN1 alone (EDTA), was necessary to effect a reduction in CD signal at 330 nm (Fig. 4, *B* and *C*). Extending these studies to the mutated proteins, we found that the addition of L97P-Ca^2+^ ions produced a decrease in the exciton pair signal, which indicated that the substrate had undergone an analogous conformational change to that when bound to WT hFEN1-Ca^2+^ (Fig. 5*A*). This reduction in signal was strictly dependent on the presence of Ca^2+^ as it is for the WT protein (Fig. 5*B*). Interestingly, the K93A/R100A double mutation also produced an altered CD signal in the presence of Ca^2+^ ions. However, a deep minima was observed, analogous to the signal detected previously with R100A and Y40A mutated hFEN1s (12). This implied that although the nucleotides positioned at −1 and −2 were reoriented in the K93A/R100A-Ca^2+^ complex, their juxtaposition at equilibrium differed from that afforded by the WT protein. In contrast, modest enhancements rather than reduction in low energy CD signals at 330 nm were observed with L111P-Ca^2+^ and L130P-Ca^2+^ when compared with the free substrate. Furthermore, the cap proline mutants afforded low energy CD signals that were very similar ± Ca^2+^ (Fig. 4, *B* and *C*). This indicated that even in the presence of divalent metal ions, L111P and L130P lacked the ability to unpair a detectable amount of the DNA substrate.

## DISCUSSION

Although biochemical and structural studies suggest a role for the conserved basic residues of the helical arch in 5′-nuclease catalysis, their precise function has remained elusive. These residues have been hypothesized to play a key role in capturing the unpaired state of substrates in FEN1 family proteins (9). In addition, the role of the hFEN1 helical cap, absent in some superfamily members, has also remained enigmatic (6, 8–10, 15–18). It has been argued that the helical cap of the protein may serve to position the required basic superfamily residues, while ensuring specificity for substrates such as flaps with 5′ termini (6, 9, 16). However, the results herein imply more complex roles for both superfamily conserved basic residues and the helical cap in the hFEN1 catalytic cycle.

In line with the observation that L97P, L111P, and L130P produce a dominant-negative phenotype in yeast, we observe negligible changes in the affinity of substrates for the mutated proteins (3). Similarly, we also demonstrate the ability to trap 5′-biotinylated substrate with streptavidin on all the mutated proteins. Moreover, each trapped complex also persisted in the presence of excess unlabeled substrate. This strongly suggests that the 5′-flap of substrate has been appropriately accommodated. Furthermore, it demonstrates that the ability to produce a trapped conformation does not require α4-α5 to adopt a defined structure. In fact, earlier experiments using 5′-flaps with duplex regions too large to pass through a structured arch demonstrate that threading the 5′-flap requires the arch to be flexible as it is in the substrate-free enzyme (15). In line with this expectation, proline mutations that grossly impact the secondary structure of α4 and α5 have negligible effects on the ability to accommodate 5′-flaps of substrates.

The substrate conformational change that hFEN1 facilitates to transfer the scissile phosphate diester to active site metal ions involves double nucleotide unpairing of duplex, a process that places the unpaired substrate within the helical arch (9, 10, 12, 19). As shown recently, Lys-93 and Arg-100 are not required for this substrate rearrangement when active site divalent metal ions are present (12). As we show here, even the introduction of proline in between these two basic residues (L97P), a mutation expected to considerably change the positioning of Lys-93 and Arg-100 side chains, does not prevent substrate unpairing. Even when both side chains are removed in K93A/R100A, DNA conformational rearrangement still occurs, although the orientation of the −1 and −2 nucleotides differs from that of DNA in WT complex. These observations rule out models where arch basic residues are required to stabilize an unpaired state. However, as these mutations to basic residues produce severely retarded reaction rates, either the substrate remains incorrectly positioned for reaction to proceed without conserved residues in place and/or the superfamily conserved basic residues are required to directly participate in catalysis.

The helical cap mutations, L111P and L130P, inhibit hFEN1 catalysis at a different catalytic step to L97P. These mutated hFEN1s are surprisingly unable to unpair any detectable amount of their DF substrates. This occurs despite evidence that divalent metal ions alter the urea-induced denaturation behavior of L111P and L130P, suggesting that the ability to bind the requisite divalent active site ions is not perturbed. In hFEN1-product structures where the hydrolyzed phosphate monoester contacts active site metal ions, the unpaired nucleobase is constrained within a cavity formed by Tyr-40 in α2 and residues from the C-terminal end of α5 (Fig. 1, *C* and *D*). However, although −1 nucleotide is stacked on Tyr-40, there are no direct helical cap-unpaired nucleobase interactions. Residue Leu-130 is at the C-terminal end of α5; as such, a proline substitution here could have disrupted the structure in this region. However, it is less straightforward, but more intriguing, to explain why the L111P mutation prevents substrate unpairing. This could be related to a role played by Leu-111 in stabilizing the two-helix cap structure through the formation of a mini hydrophobic α4-α5 helix-helix core. Moreover, α5 packs against α2 and the α2-α3 loop when substrate is present. These same regions of the protein are partly disordered in structures without DNA (Fig. 1, *B* and *C*), but form interactions with substrate when present. An altered helical cap region could sterically exclude substrate unpairing by preventing accommodation of the 5′-flap through the arch; however, the ability to form trapped complexes with these mutants suggests that the 5′-flap of the substrate can pass under the helical cap, at least while duplex remains in a paired state. Alternatively, the inability to rearrange the DNA close to active site metal ions suggests a role for the helical cap in formation of unpaired DNA, either directly by the requirement to restrain one or both of the unpaired nucleobases and/or by participation in protein conformational changes necessary to afford the unpaired state.

Interestingly, although K93A and R100A independently have large impacts on the ability to catalyze the reaction, the double mutant K93A/R100A is no more detrimental to catalysis than R100A alone. This indicates cooperation between the basic residues of the active site because loss of Arg-100 interaction impacts on the ability of Lys-93 to participate in the reaction. A similar phenomenon has also been observed independently in time-resolved fluorescence studies of 2AP substrates. Interaction of FEN1-conserved Tyr-40 of α2 with the −1 nucleobase could not be detected in R100A and K93A complexes (12). Despite this evidence for the cooperative assembly of conserved residues around unpaired DNA, the impact of the secondary structure altering L97P is notably much greater than those of removal of the side chains of the basic conserved residues Lys-93 and Arg-100. This implies that a lack of these interactions is not the only consequence of L97P mutation. It would appear that conformational rigidity of proline locks the hFEN1-DNA complex into a conformation that is catalytically compromised in either a substrate-unpaired state (L97P) or a base-paired state (L111P and L130P).

The finding that perturbation of the helical cap can prevent double nucleotide unpairing of substrate raises questions about superfamily members XPG and GEN1, which lack this feature. These enzymes have specificities for reaction one nucleotide into the double strand of their respective substrates, bubbles, and four-way-junctions, suggesting that they also unpair DNA. It seems likely that these larger enzymes or their partner proteins make alternative interactions to position unpaired DNA within the active site. The addition of an archaeal helical cap together with removal of the 650-residue spacer domain of XPG did confer the ability to process substrates with free 5′ termini to the XPG-FEN1 chimera XPF2 (9). Unlike bubble-incising XPG, XFX2 is a reasonably proficient flap endonuclease, although it lacks the single scissile phosphate specificity of FEN1.

In conclusion, the helical cap is revealed to have a more complex role in the hFEN1 catalytic cycle in addition to its roles in positioning of essential residues or acting as a barrier to DNA substrates without 5′ termini. A role for substrate unpairing in threading flaps under the cap is ruled out by our studies; instead, 5′-flap accommodation, which does not require a unique helical arch conformation, must precede DNA conformational change. Unpairing does not require conserved basic residues, although they are essential to catalyze reactions on a biologically relevant time scale. It is interesting that despite a requirement to unpair DNAs, hFEN1 does not process substrates with non-Watson Crick base pairs at the end of reacting duplexes efficiently (13, 19). Indeed, hFEN1 appears to initially stabilize the base-paired state by stacking interactions with α2, thereby selecting against substrates with frayed ends. There is an intriguing association between an intact helical cap and substrate conformational change, alongside an unusually low stability of the protein to denaturant and the ability of rigid proline mutations to inhibit reaction. Altogether, these data suggest that conformational dynamics of hFEN1-substrate complexes are likely to play a role in creating the unpaired state.

## Footnotes

7 The abbreviations used are:

FEN1: flap endonuclease-1
hFEN1: human FEN1
EXO1: exonuclease-1
GEN1: gap endonuclease-1
2AP: 2-amino purine
DF: double-flap
SA: streptavidin
XPG: xeroderma pigmentosum complementation group G.

## References

[1] Finger L. D. Blanchard M. S. Theimer C. A. Sengerová B. Singh P. Chavez V. Liu F. Grasby J. A. Shen B. (2009) The 3′-flap pocket of human flap endonuclease 1 is critical for substrate binding and catalysis. J. Biol. Chem. 284, 22184–22194 19525235 10.1074/jbc.M109.015065 PMC2755943

[2] Lyamichev V. Brow M. A. Varvel V. E. Dahlberg J. E. (1999) Comparison of the 5′ nuclease activities of *Taq* DNA polymerase and its isolated nuclease domain. Proc. Natl. Acad. Sci. U.S.A. 96, 6143–6148 10339555 10.1073/pnas.96.11.6143 PMC26849

[3] Storici F. Henneke G. Ferrari E. Gordenin D. A. Hübscher U. Resnick M. A. (2002) The flexible loop of human FEN1 endonuclease is required for flap cleavage during replication and repair. EMBO J. 21, 5930–5942 12411510 10.1093/emboj/cdf587 PMC131084

[4] Williams R. Sengerová B. Osborne S. Syson K. Ault S. Kilgour A. Chapados B. R. Tainer J. A. Sayers J. R. Grasby J. A. (2007) Comparison of the catalytic parameters and reaction specificities of a phage and an archaeal flap endonuclease. J. Mol. Biol. 371, 34–48 17559871 10.1016/j.jmb.2007.04.063 PMC1993357

[5] Finger L. D. Atack J. M. Tsutakawa S. Classen S. Tainer J. Grasby J. Shen B. (2012) The wonders of flap endonucleases: structure, function, mechanism, and regulation. in The Eukaryotic Replisome: A Guide to Protein Structure and Function (MacNeill S., ed), pp. 301–326, Springer Publishing, New York 10.1007/978-94-007-4572-8_16 PMC3728657 22918592

[6] Grasby J. A. Finger L. D. Tsutakawa S. E. Atack J. M. Tainer J. A. (2012) Unpairing and gating: sequence-independent substrate recognition by FEN superfamily nucleases. Trends Biochem. Sci. 37, 74–84 22118811 10.1016/j.tibs.2011.10.003 PMC3341984

[7] Syson K. Tomlinson C. Chapados B. R. Sayers J. R. Tainer J. A. Williams N. H. Grasby J. A. (2008) Three metal ions participate in the reaction catalyzed by T5 flap endonuclease. J. Biol. Chem. 283, 28741–28746 18697748 10.1074/jbc.M801264200 PMC2568906

[8] Tomlinson C. G. Atack J. M. Chapados B. Tainer J. A. Grasby J. A. (2010) Substrate recognition and catalysis by flap endonucleases and related enzymes. Biochem. Soc. Trans. 38, 433–437 20298197 10.1042/BST0380433

[9] Tsutakawa S. E. Classen S. Chapados B. R. Arvai A. S. Finger L. D. Guenther G. Tomlinson C. G. Thompson P. Sarker A. H. Shen B. Cooper P. K. Grasby J. A. Tainer J. A. (2011) Human flap endonuclease structures, DNA double-base flipping, and a unified understanding of the FEN1 superfamily. Cell 145, 198–211 21496641 10.1016/j.cell.2011.03.004 PMC3086263

[10] Orans J. McSweeney E. A. Iyer R. R. Hast M. A. Hellinga H. W. Modrich P. Beese L. S. (2011) Structures of human exonuclease 1 DNA complexes suggest a unified mechanism for nuclease family. Cell 145, 212–223 21496642 10.1016/j.cell.2011.03.005 PMC3093132

[11] Devos J. M. Tomanicek S. J. Jones C. E. Nossal N. G. Mueser T. C. (2007) Crystal structure of bacteriophage T4 5′ nuclease in complex with a branched DNA reveals how FEN-1 family nucleases bind their substrates. J. Biol. Chem. 282, 31713–31724 17693399 10.1074/jbc.M703209200

[12] Finger L. D. Patel N. Beddows A. Ma L. Exell J. C. Jardine E. Jones A. C. Grasby J. A. (2013) Observation of unpaired substrate DNA in the flap endonuclease-1 active site. Nucleic Acids Res. 10.1093/nar/gkt737 PMC3834815 23975198

[13] Beddows A. Patel N. Finger L. D. Atack J. M. Williams D. M. Grasby J. A. (2012) Interstrand disulfide crosslinking of DNA bases supports a double nucleotide unpairing mechanism for flap endonucleases. Chem. Comm. 48, 8895–8897 22850542 10.1039/c2cc33400c

[14] Sakurai S. Kitano K. Yamaguchi H. Hamada K. Okada K. Fukuda K. Uchida M. Ohtsuka E. Morioka H. Hakoshima T. (2005) Structural basis for recruitment of human flap endonuclease 1 to PCNA. EMBO J. 24, 683–693 15616578 10.1038/sj.emboj.7600519 PMC549611

[15] Patel N. Atack J. M. Finger L. D. Exell J. C. Thompson P. Tsutakawa S. Tainer J. A. Williams D. M. Grasby J. A. (2012) Flap endonucleases pass 5′-flaps through a flexible arch using a disorder-thread-order mechanism to confer specificity for free 5′-ends. Nucleic Acids Res. 40, 4507–4519 22319208 10.1093/nar/gks051 PMC3378889

[16] Gloor J. W. Balakrishnan L. Bambara R. A. (2010) Flap endonuclease 1 mechanism analysis indicates flap base binding prior to threading. J. Biol. Chem. 285, 34922–34931 20739288 10.1074/jbc.M110.165902 PMC2966106

[17] Ceska T. A. Sayers J. R. Stier G. Suck D. (1996) A helical arch allowing single-stranded DNA to thread through T5 5′-exonuclease. Nature 382, 90–93 8657312 10.1038/382090a0

[18] Murante R. S. Rust L. Bambara R. A. (1995) Calf 5′ to 3′ exo/endonuclease must slide from a 5′ end of the substrate to perform structure-specific cleavage. J. Biol. Chem. 270, 30377–30383 8530463 10.1074/jbc.270.51.30377

[19] Sobhy M. A. Joudeh L. I. Huang X. Takahashi M. Hamdan S. M. (2013) Sequential and multistep substrate interrogation provides the scaffold for specificity in human flap endonuclease 1. Cell Reports 3, 1785–1794 23746444 10.1016/j.celrep.2013.05.001

[20] Tock M. R. Frary E. Sayers J. R. Grasby J. A. (2003) Dynamic evidence for metal ion catalysis in the reaction mediated by a flap endonuclease. EMBO J. 22, 995–1004 12606565 10.1093/emboj/cdg098 PMC150332

[21] Patel D. Tock M. R. Frary E. Feng M. Pickering T. J. Grasby J. A. Sayers J. R. (2002) A conserved tyrosine residue aids ternary complex formation but not catalysis, in phage T5 flap endonuclease. J. Mol. Biol. 320, 1025–1035 12126622 10.1016/s0022-2836(02)00547-8

[22] Kelly S. M. Jess T. J. Price N. C. (2005) How to study proteins by circular dichroism. Biochim. Biophys. Acta 1751, 119–139 16027053 10.1016/j.bbapap.2005.06.005

[23] Warren J. R. Gordon J. A. (1966) On the refractive indices of aqueous solutions of urea. J. Phys. Chem. 70, 297–300

[24] Clarke J. Fersht A. R. (1993) Engineered disulfide bonds as probes of the folding pathway of barnase: increasing the stability of proteins against the rate of denaturation. Biochemistry 32, 4322–4329 8476861 10.1021/bi00067a022

[25] Sengerová B. Tomlinson C. Atack J. M. Williams R. Sayers J. R. Williams N. H. Grasby J. A. (2010) Brønsted analysis and rate-limiting steps for the T5 flap endonuclease catalyzed hydrolysis of exonucleolytic substrates. Biochemistry 49, 8085–8093 20698567 10.1021/bi100895j

[26] Tomlinson C. G. Syson K. Sengerová B. Atack J. M. Sayers J. R. Swanson L. Tainer J. A. Williams N. H. Grasby J. A. (2011) Neutralizing mutations of carboxylates that bind metal 2 in T5 flap endonuclease result in an enzyme that still requires two metal ions. J. Biol. Chem. 286, 30878–30887 21734257 10.1074/jbc.M111.230391 PMC3162448

[27] Fersht A. (1999) Structure and Mechanism in Protein Science: Guide to Enzyme Catalysis and Protein Folding, 2nd Ed., W.H. Freeman & Co Ltd

